# Robot-assisted treatment of secondary epilepsy caused by parasitic infection: a case report

**DOI:** 10.1186/s42494-024-00161-8

**Published:** 2024-05-15

**Authors:** Juan Luo, Xin Chen, Sixun Yu, Haifeng Shu

**Affiliations:** 1 Department of Neurosurgery, Affiliated Hospital of Southwest Medical University, Luzhou Sichuan, 646000 China; 2 Department of Neurosurgery, General Hospital of the Western Theater Command of the PLA, Chengdu Sichuan, 610083 China

**Keywords:** Cerebral sparganosis, Secondary epilepsy, Robot, Frameless stereotaxic technology

## Abstract

**Background:**

Cerebral sparganosis represents the most severe manifestation of sparganosis, with a relatively low global incidence. For cases of secondary epileptic seizures caused by sparganosis infection in the functional areas of the brain, what advanced neurosurgical techniques should be employed to precisely identify and excise the epileptic lesions in the deep functional areas of the brain, aiming to achieve maximal removal while minimising the risk of neurological deficits? This remains a current challenge for epilepsy surgeons.

**Case presentation:**

A 24-year-old Chinese male was admitted to our hospital, presenting with a history of left limb twitching persisting for over a year. His main clinical symptoms presented twitching and numbness of his left limb without loss of consciousness. Under the premise of inappropriate anti-seizure treatment, recurrent epilepsy attacked persist. The patient's diagnosis was considered as “space-occupying lesions in the several lobes of brain, secondary epilepsy” after comprehensive assessment and discussion. And experts considered that the patient's space-occupying lesions in the right frontal and parietal lobes were highly suspected to be infected by parasites. This report delved into the application of neurosurgery robot-assisted frameless stereotaxic technology and intraoperative stereotactic electroencephalography (SEEG) monitoring technology to accurately locate and optimize removal of parasite-related epileptic lesions situated in functional areas of the brain. As a result, the patient had achieved seizure freedom, leaving no symptoms of neurological deficit.

**Conclusions:**

With the highly integrated development of imaging technology, mechanical technology, computer control technology, and artificial intelligence, surgical robots are poised to play a larger role across various neurosurgical specialties in the future. Considering benefits for patients and the promising application of this technology, its utilization holds significant value.

**Supplementary Information:**

The online version contains supplementary material available at 10.1186/s42494-024-00161-8.

## Background

Sparganosis is a zoonotic taenia infection caused by the plerocercoid or second-stage larvae (sparganum) of pseudophyllidean tapeworms that reside in host tissues [1]. Upon entering the human body, sparganum usually establish parasitic infection and induce lesions in the subcutaneous tissue or muscle tissue. However, a minority of sparganum can also invade the human brain, resulting in chronic active inflammation subsequent to central nervous system (CNS) invasion. Which leads to a series of neurological symptoms such as epileptic seizures, sensory disturbances, and headaches in patients, known as cerebral sparganosis. Cerebral sparganosis accounts for approximately 25% of all sparganosis cases [2]. The primary mode of infection is through the digestive tract, such as drinking water contaminated with water fleas or eating raw or undercooked intermediate hosts like frog, snake or chicken meat [3–6]. Cerebral sparganosis is a very rare zoonotic disease which is regarded as the most severe manifestation of sparganosis [7, 8]. Seizures are frequently reported as the most common symptom in patients with cerebral sparganosis [9]. This article presents a case of secondary epilepsy caused by cerebral sparganosis and focuses on the application of neurosurgery robot-assisted frameless stereotaxic technology and intraoperative stereotactic electroencephalography (SEEG) monitoring, aiming to precisely identify lesions within vital brain regions and optimize surgical excision [10–13]. Successful resection of lesions in functional areas of the brain is expected to achieve seizure freedom without leaving neurological deficits. In addition, we were lucky enough to identify and extract the three live worm bodies of sparganum in the brain through neurosurgical robot-assisted frameless stereotactic technology, thereby prompting the reporting of this unique case.

### Case presentation

A 24-year-old male patient was admitted to the hospital on November 26, 2021, presenting with a history of left limb twitching persisting for over a year. In August 2020, the patient had the first seizure onset attack, which manifested as sudden paroxysmal left upper arm rigidity without any identifiable trigger and loss of consciousness, accompanied by numbness and a sensation feeling of crawling ants in the left upper arm, along with visual field loss, lasting about 1 min. However, the patient and his family did not pay attention to the symptons, and consequently no medical intervention was sought at that time. From September to October in 2020, the patient experienced similar seizures attack for five times, accompanied by visual field defect. Each episode lasted approximately 1 min before the convulsions subsided. Additionally, the patient reported experiencing a headache lasting for about 1 h following the seizures. On November 10, 2021, the patient had another seizure without an apparent trigger while awake. This seizure was presented with twitching in the left limb (including upper and lower limbs), backward tilting of the head, eyes gazing to the left, and the corner of the mouth tilting to the left. Concurrent symptoms included numbness and weakness of the left limb, visual field defect, palpitation, nausea, and profuse sweating. The symptoms relieved after about 2 min, but the ensuing headache persisted for several hours before gradually relieving. The patient was subsequently sent to a local hospital for treatment. His head magnetic resonance imaging (MRI) scan revealed abnormal signal shadows in the right precentral gyrus, leading to the diagnosed of secondary epilepsy. At the beginning, the patient followed the doctor's advice and initiated a regimen of sodium valproate sustained-release tablets 0.5 g bid for a week, then he gradually reduced it untill stopped taking the medicine by himself. On November 26, 2021, the similar seizure pattern relapsed, but this time there were 5 consecutive bead-like seizures (cluster seizures), occurring approximately 2–3 min apart from each other. The patient spontaneously recovered after half an hour, remaining conscious throughout the episode. Subsequently, the patient was promptly sent to the emergency department of our hospital for medical treatment. The emergency electrocardiogram (ECG) showed that the QT interval was normal. Considering the patient's secondary epilepsy diagnosis and the history of frequency seizures, a temporarily prescription of sodium valproate sustained-release tablets 0.5 g bid was administered. Additionally, the patient was recommended to be transferred to our department for further diagnosis and treatment.

**Fig. 1:**
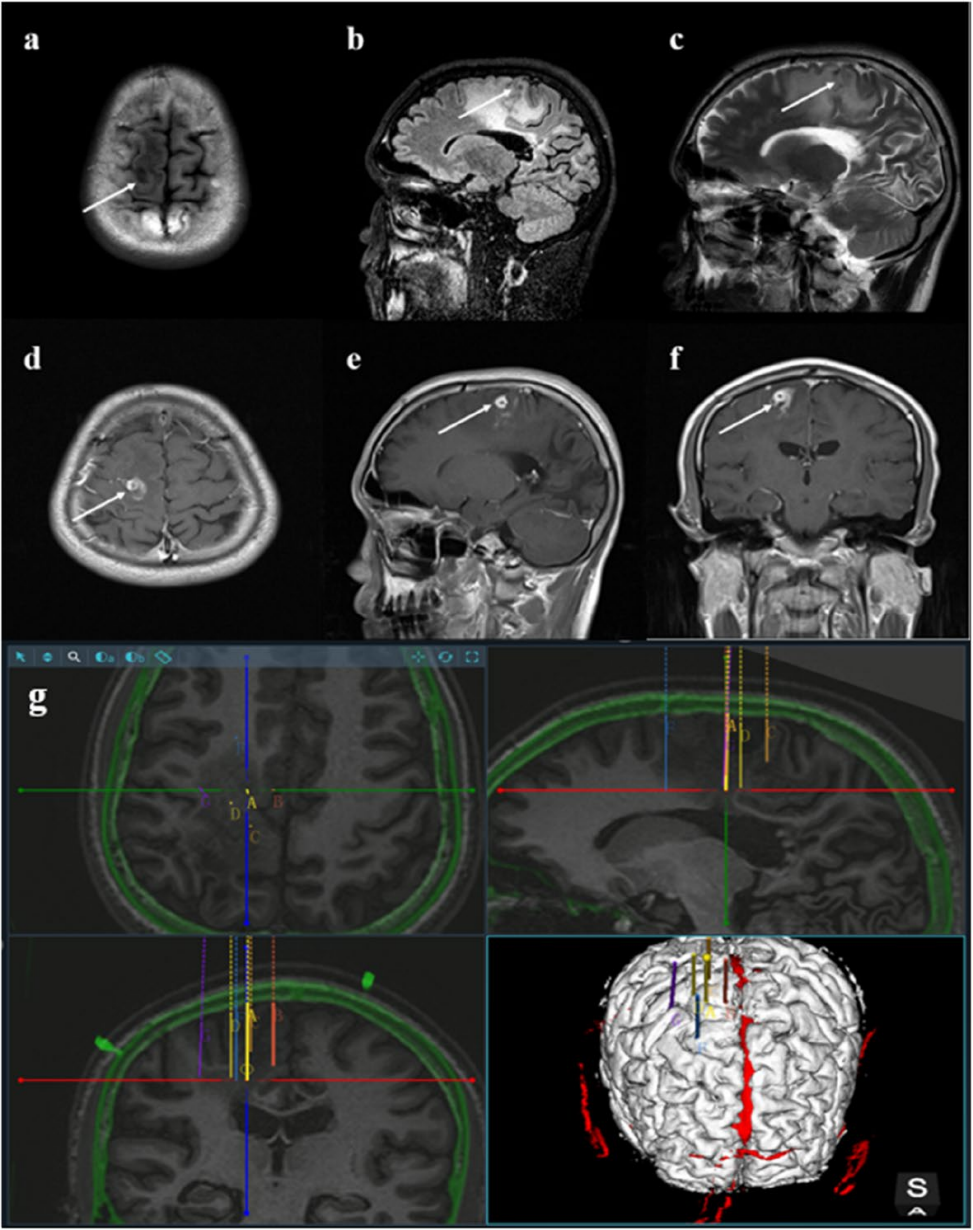
Preoperative MRI images in axial (**a**) T1-weighted, sagittal (**b**) T2/FLAIR-weighted, and sagittal (**c**) T2-weighted scans. Preoperative contrast-enhanced T1-weighted MRI images in the axial (**d**), sagittal (**e**), and coronal (**f**) planes are presented. Neurosurgery robot-assisted frameless stereotactic technology was applied to implement the surgical strategy for lesion biopsy and resection in functional areas (**g**)

**Fig. 2:**
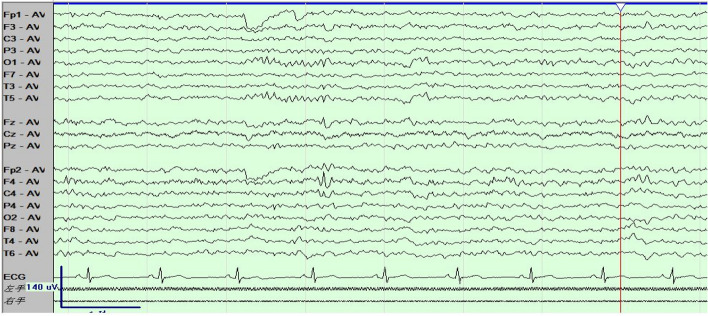
The video electroencephalogram (VEEG) recordings of the patient

Throughout the patient's hospitalization in our department, the majority of laboratory tests and cerebrospinal fluid analysis yielded normal results. However, the patient did indeed exhibit motor paralysis in the left upper limb, as evidenced by a score of 3 out of 5 on the manual muscle test (MMT). The plain cerebral MRI scan showed the presence of patchy mixed signal shadows in the right fronto-parietal lobe, particularly in the precentral gyrus area. These shadows were characterized by slightly prolonged T1 and T2 signals, mixed with patchy areas of short T2 signals. Additionally, a significant area of edema was observed surrounding these lesions. In the right parietal and occipital lobes, some lesions exhibited low signal intensity while others displayed high signal intensity, indicative of potential softening lesions (Fig. 1a–c). Enhanced brain MRI imaging revealed a heterogeneous nodular mixed signal shadow in the right precentral gyrus, surrounded by a large area of edema, suggesting a potential presence of space-occupying lesion in this area. Some lesions in the right parieto-occipital lobe showed low signal intensity, while other lesions showed high signal intensity, indicating the possibility of softening lesions forming in the right parieto-occipital lobe (Fig. 1d–f). The video electroencephalogram (VEEG) results showed the occasional presense of atypical sharp wave components with medium amplitude (46–78 µV) around 5.4 Hz in the right frontal area during light sleep, but no abnormalities were detected in the rest (Fig. 2). Thorough questioning of the patient's history revealed that he had the habit of consuming raw food during his time working in the coastal area of Guangdong Province. Additionally, the patient had suffered trauma to his right occipital region over a decade ago. Following a comprehensive multimodal assessment and multidisciplinary discussion, the patient's diagnosis was considered as “1.space-occupying lesions in the right frontal lobes and parietal lobes; 2.space-occupying lesions in the right occipital lobe; 3.secondary epilepsy: Jacksonian epilepsy without disturbance of consciousness”. The medical team considered that the space-occupying lesion present on the patient's right frontal and parietal lobe was highly suspected to be caused by parasitic infection. Furthermore, the space-occupying lesion in the right occipital lobe was identified as the underlying cause of the patient's visual field defect, which may stem from a previous brain trauma during his adolescence. Our team provided a detailed explanation of the patient’s condition, feasible treatment options and related risks to the patient and his family. The primary concern for the patient is to solve the problem of the recurrence of epilepsy. The critical approach to addressing this issue involved accurate localization and maximal resection of epileptic lesions within the functional brain areas, while minimizing unnecessary damage to surrounding brain tissue. Meanwhile, two surgical methods: traditional craniotomy to remove lesions or stereotactic intracranial lesion biopsy technology were provided to patient and his families. We also recommended the patient for further parasite-specific testing identify etiology, but this proposal was rejected. Finally, the patient and his families chose stereotaxic intracranial lesion biopsy technology including framed and frameless stereotactic techniques [14]. Frameless stereotactic technology consists of two main types: neurosurgical navigation systems and neurosurgical robots [10–13]. Our epilepsy team utilized neurosurgery robot-assisted frameless stereotaxic technology along with intraoperative SEEG monitoring technology. The neurosurgical robot used in the operation utilizes the Huake Precision® SR1-3D independently developed by Huake Precision (Beijing) Medical Technology Co., Ltd, using 3D structured light technology and HoloShot intelligent sensing technology to provide neurosurgeons with precise, convenient and effective surgical assistance during the operation. The surgical plan (Fig. 1g) and surgical procedures are generally summarized as follows. In the preoperative preparation stage, three steps were involved: (1) replicated the patient’s preoperative head CT, MRI and other imaging examination information to formulate a surgical plan; (2) inserted 5 metal markers at different positions on the patient's skull, performed a thin-slice CT positioning scan with a slice thickness of 1–2 mm, and copied the acquired image information to the neurosurgery robot computer workstation in the operating room; (3) the epilepsy surgery team utilized the “surgical treatment planning” software on the neurosurgical robot’s computer to integrate the surgical plan and thin-slice CT images information through multi-modal image fusion for accurate positioning. During the operation, the specific surgical steps were as follows. (1) The patient was brought into the operating room and placed supine on the operating bed, where he underwent general anesthesia. The epilepsy surgeon used a head frame to fix the patient's head on the operating bed, and then connected and fixed the position between the patient's operating bed and the neurosurgery robot. Subsequently, the team used the robotic arm working platform of the neurosurgical robot to register and matched the markers, outlining the lesion and creating a three-dimensional visualization of the target path. This process involved obtaining the three-dimensional coordinates of the target (X, Y, Z), calculating the optimal cranial entry point and biopsy trajectory, as well as determining the precise location of the target coordinates. (2) Following the determination of the optimal cranial entry point coordinates, the surgeon designed a linear incision centered on the right fronto-parietal lobe lesion measuring about 5 cm in length. After routine disinfection and draping procedures, the galea aponeurosis of the scalp was incised in layers. Bipolar electrocoagulation was employed to stop bleeding, and a spreader was used to expand the skin and muscle flaps for the skull exposure. A hole was then drilled into the skull to form a circular bone flap, which was subsequently removed to form a 4*3 cm bone window. After the bone flap being extracted, the cross-shaped incision was made in the dura mater. (3) The surgeon determined the lesion and the cortex at the surgical site according to the robot's positioning system. Initially, deep electrodes were utilized for intraoperative SEEG monitoring, revealing the presence of a small amount of sharp waves deep within the brain tissue of the surgical area. Following the monitoring, brain cotton pads were used to protect brain tissue beyond the precentral sulcus. (4) Bipolar electrocoagulation and a nerve dissector were utilized to create a cortical fistula for exploration of the lesion center. About 0.5 cm below the cortex, the brain tissue exhibited white and relatively firm texture. The nerve dissector was employed to identify the clear boundary between the surrounding and normal white matter. Carefully dissection along this boundary allowed for the separation of a small round nodule from the lesion. The capsule of this nodule was visible, after it being sent for pathological examination, the surgen continued the exploration. Surprisingly, three live milky-white parasites were extracted from the lesion and its surroundings, the longest of which was 12 cm in length (see Video 1 s in the online-only Supplementary material for a living intracranial parasite.). Exploring to a depth of approximately 3 cm below the cortex and microscopic examination, the lesion had been completely excised, and the parasites along with the affected tissue were sent for pathological analysis to identify the nature. Subsequent electrophysiological monitoring showed a return to basically normal brain activity in the surgical area. A large amount of normal saline and an appropriate amount of hemostatic gauze were used to flush and packed. (5) Cranial closure operation was complete.

**Fig. 3:**
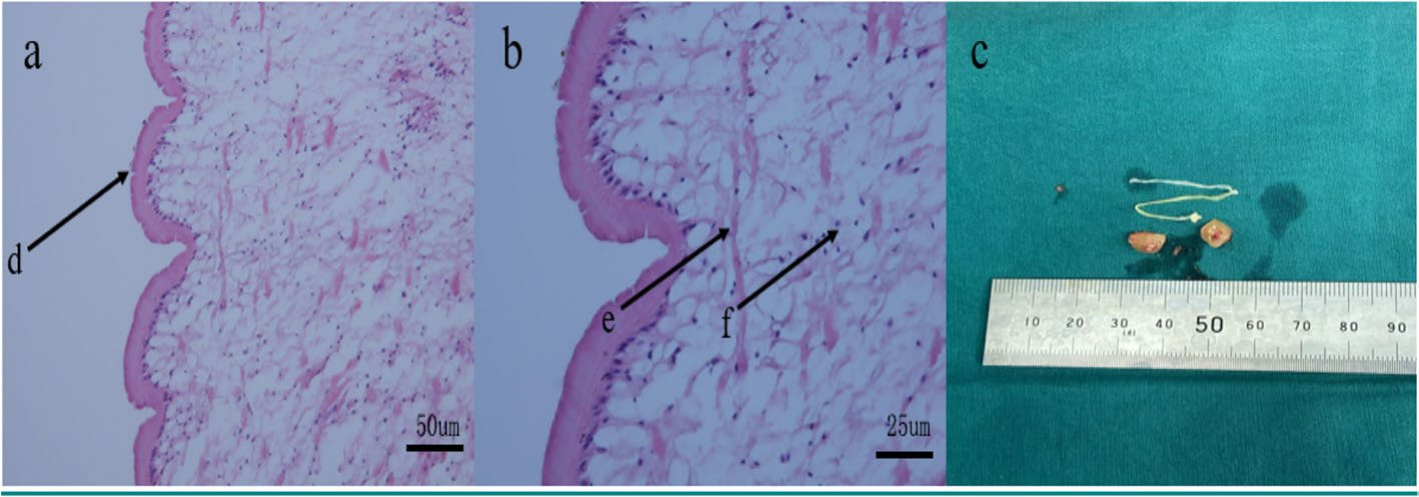
The pathological results of the parasite body are consistent with the performance of sparganosis. **a**. The d arrow points to the structure of different sections of the insect body, body surface transverse stripes (H&E, × 200); **b**. The e arrow points to the longitudinal muscles and the f arrow points to the calcerous body (H&E, × 400). **c**. The first living sparganum were captured

**Fig. 4:**
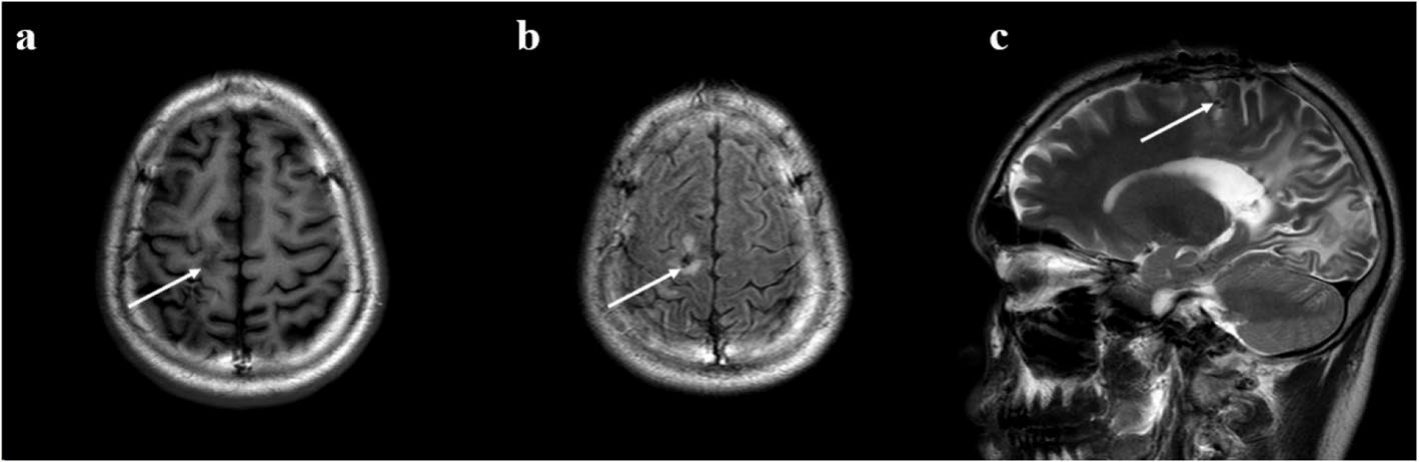
Postoperative follow-up brain MRI scans

**Fig. 5:**
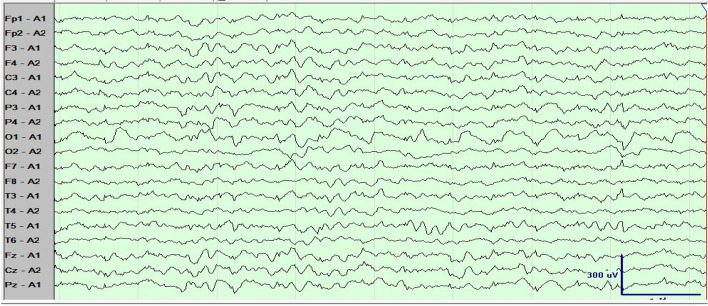
The VEEG recording of the patient 2 years after surgery showed that no abnormal intracranial discharges in any brain region during the monitoring process

The subsequent pathological results revealed as follows: (1) the diseased brain tissue showed nerve tissue and inflammatory necrotic components, with eosinophilic abscess formation, fibrosis, and encapsulation; (2) the parasitic worms were identified as sparganum (Fig. 3a, b). Additionally, the morphology of the parasite body also supports this diagnosis (Fig. 3c). Then, the patient was prescribed praziquantel for anthelmintic therapy and anti-seizure medication (ASM) sodium valproate sustained-release tablets 0.5 g bid. In the 2-year follow-up, the patient demonstrated a mini-mental state examination (MMSE) scale score of 29, and a Montreal cognitive assessment (MOCA) scale score of 28, indicating no post-surgery cognitive dysfunction. The score of Hamilton depression rating scale (17 items) was 1, and the score of Hamilton anxiety rating scale (14 items) was 3, indicating no post-surgery depression or anxiety. In the emotional-social loneliness questionnaire, the isolation score was 5, the emotional loneliness score was 3, and the social loneliness score was 0. Therefore, it was considered that the patient had no or almost no isolation, emotional loneliness, and social loneliness after surgery. The patient's quality of life in epilepsy inventory-31 (QOLIE-31) scale score was 89.47. The cerebral MRI plain scans (Fig. 4a, b) were performed 2 years after the operation, revealing complete removal of the right frontal and parietal epileptic lesions without the emergence of new similar brain lesions. Additionally, significant reduction in edema surrounding the epileptic lesion was observed. The patient's right occipital lobe softening was considered to be caused by brain trauma, which was also the reason for his vision impairment, so this change still existed in the follow-up MRI. The VEEG recordings showed that no abnormal intracranial discharges in any brain region during the monitoring process (Fig. 5). During regularly 2-year flow-up in the outpatient department of our hospital's epilepsy center, the patient became seizure-free. Moreover, the subsequent ASM adjustment plan for the patient was to gradually reduce until stop taking medicine.

## Discussion

In this case, the delayed diagnosis was attributed to the rarity of cerebral sparganosis and the technical limitations of primary hospitals. Later, the detailed inquiry into the patient's history revealed a previous habit of eating raw food when he worked in the coastal area. So our epilepsy center team further improved the patient's multimodal assessment examination. Based on the findings from the multimodal assessment and following multidisciplinary deliberations, it was suspected a possible correlation between the epileptic seizures and tapeworm infection. At that time, the challenge faced by our team was precisely identifying and excising the epileptic lesions located in the functional brain area to the greatest extent. To address this challenge, our team had leveraged neurosurgery robot-assisted technology and used frameless stereotaxic technology. The results indicated the patient achieved seizure-freedom with no neurological deficits. Hence, our team advocates the expanded application of robot-assisted surgery in cases where surgical resection of deep-seated epileptic lesions within functional brain areas. This approach is deemed valuable from the perspective of patient benefits and the potential application prospects of this technology.

In contrast to traditional craniotomy surgery, neurosurgery robot-assisted technology also presents distinct advantages and certain limitations. The advantages of robot-assisted technology can be summarized in three aspects [11]: (1) enhancing surgical accuracy, minimizing human errors, and bolstering operative safety; (2) reducing surgical duration, enhancing operational efficiency, and alleviating physician fatigue; (3) automating the generation and simulation of optimal surgical plans, thereby simplifying and streamlining the surgical process. However, it also has some limitations. For instance, the high cost of equipment may hinder its popularity in some regions and hospitals; it requires doctors to possess a certain level of technical proficiency and extensive experience; the current indications are relatively limited and it mainly focuses on some specific surgeries [15], such as brain biopsy and deep brain hemorrhage [15–17]. Additionally, this technology still encounters several challenges, including technical complexity, operator learning curve, and patient acceptance of the new technology. In this case, our department has already possessed neurosurgical robotic instruments, and the epilepsy surgery team has demonstrated proficient and efficiency in mastering this technology. Moreover, as this technology has been maturely implemented within our department, patients have become more receptive to the expanded application of it. In this case, the surgeon could only rely on visual feedback to perform relevant surgical operations during the operation, consequently increasing the risks and uncertainties. To address this issue, future advancements can involve the development of novel control algorithms aiming to regulate the human-robot interaction. Similar to the electronic real-time navigation broadcast system in cars, these algorithms can provide real-time guidance to surgeons, indicating whether the surgical trajectory is accurate and aiding in the decision-making process. The expert team can also alleviate the challenges associated with this technology by improving accuracy, optimizing the surgical process, and reducing the complexity of the surgery, which aim to serve patients better. The cost-effectiveness of robot-assisted surgery has been confirmed by several studies [15, 18]. However, the accessibility of this technology remains restrained, particularly in areas where it may not be easily accessible. Therefore, further researches and policy supports are essential to enhance the accessibility of this technology. Considering current development trends, future research directions could entail the development of innovative device designs that prioritize precision to enhance the automation of stereotaxy [19]. For example, developing robots in the form of exoskeletons and creating intent detection methods can improve interaction experience and work efficiency. Furthermore, as imaging technology, mechanical technology, computer control technology, and artificial intelligence become increasingly integrated, the progression of surgical robot technology is poised to escalate significantly. In the future, surgical robots are anticipated to be employed in a broader spectrum of neurosurgical specialties, improving the safety and efficiency of surgical procedures [20]. These developments will bring new opportunities for the clinical implementation of robot-assisted surgery.

## Conlusions

With this case, our epilepsy team has expanded knowledge about cerebral sparganosis. In addition, it is worth mentioning that in this case, for patients with symptomatic epilepsy caused by cerebral sparganosis, the proficient use of frameless stereotaxic neurosurgical robot-assisted technology and collaborative teamwork can be extremely helpful. It allows for accurate identification and removal of epileptic lesions, leading to good outcomes such as achieving seizure freedom without neurological deficits.

## Supplementary Information

**Additional file 1: Video.1s.**  A living intracranial parasite.

## Data Availability

The datasets used and/or analysed in this case report are available from the corresponding author on reasonable request.

## Abbreviations

ASM: Anti-seizure medication
CNS: Central nervous system
ECG: Electrocardiogram
MMSE: Mini-mental state examination
MMT: Manual muscle test
MOCA: Montreal cognitive assessment
MRI: Magnetic resonance imaging
QOLIE-31: Quality of life in epilepsy inventory-31
SEEG: Stereotactic electroencephalography
VEEG: Video electroencephalogram

## References

[1] Woldemeskel M. Subcutaneous sparganosis, a zoonotic cestodiasis, in two cats. J Vet Diagn Invest. 2014;26(2):316–9. 24464556 10.1177/1040638713517697

[2] Liu W, Gong T, Chen S, Liu Q, Zhou H, He J, et al. Epidemiology, Diagnosis, and Prevention of Sparganosis in Asia. Animals (Basel). 2022;12(12):1578. 35739914 10.3390/ani12121578 PMC9219546

[3] Yu Y, Shen J, Yuan Z, Xia Z, Gao F, Jiang L, et al. Cerebral Sparganosis in Children: Epidemiologic and Radiologic Characteristics and Treatment Outcomes: A Report of 9 Cases. World neurosurgery. 2016;89:153–8. 26855309 10.1016/j.wneu.2016.01.086

[4] Xie X, Hu J, Sun G, Ding B, Feng L. Orbital sparganosis in an 8-year boy: a case report. BMC Ophthalmol. 2018;18(1):13. 29357839 10.1186/s12886-018-0675-8 PMC5778690

[5] Nkwerem S, Goto T, Ogiwara T, Yamamoto Y, Hongo K, Ohaegbulam S. Ultrasound-Assisted Neuronavigation-Guided Removal of a Live Worm in Cerebral Sparganosis. World Neurosurg. 2017;102:696.e13-e16. 28315796 10.1016/j.wneu.2017.03.031

[6] Chen X, Wu H, Lu L, Zhou N, Chen Z, Zhang X. Cerebral sparganosis in a child with corpus callosum invasion: a case report. BMC Infect Dis. 2023;23(1):350. 37231358 10.1186/s12879-023-08322-9 PMC10210387

[7] Zhu Y, Ye L, Ding X, Wu J, Chen Y. Cerebral sparganosis presenting with atypical postcontrast magnetic resonance imaging findings: a case report and literature review. BMC Infect Dis. 2019;19(1):748. 31455261 10.1186/s12879-019-4396-2 PMC6712767

[8] Nobayashi M, Hirabayashi H, Sakaki T, Nishimura F, Fukui H, Ishizaka S, et al. Surgical removal of a live worm by stereotactic targeting in cerebral sparganosis. Case report Neurologia medico-chirurgica. 2006;46(3):164–7. 16565589 10.2176/nmc.46.164

[9] Hong D, Xie H, Zhu M, Wan H, Xu R, Wu Y. Cerebral sparganosis in mainland Chinese patients. J Clin Neurosci: official journal of the Neurosurgical Society of Australasia. 2013;20(11):1514–9. 10.1016/j.jocn.2012.12.018 23911107

[10] Yasin H, Hoff HJ, Blümcke I, Simon M. Experience with 102 Frameless Stereotactic Biopsies Using the neuromate Robotic Device. World neurosurgery. 2019;123:e450–6. 30500594 10.1016/j.wneu.2018.11.187

[11] Terrier L, Gilard V, Marguet F, Fontanilles M, Derrey S. Stereotactic brain biopsy: evaluation of robot-assisted procedure in 60 patients. Acta Neurochir. 2019;161(3):545–52. 30675655 10.1007/s00701-019-03808-5

[12] Legnani FG, Franzini A, Mattei L, Saladino A, Casali C, Prada F, et al. Image-Guided Biopsy of Intracranial Lesions with a Small Robotic Device (iSYS1): A Prospective, Exploratory Pilot Study. Operative neurosurgery (Hagerstown, Md). 2019;17(4):403–12. 30690491 10.1093/ons/opy411

[13] Liu HG, Liu YY, Zhang H, Meng FG, Zhang K, Zhu GY, et al. A Bulk Retrospective Study of Robot-Assisted Stereotactic Biopsies of Intracranial Lesions Guided by Videometric Tracker. Front Neurol. 2021;12: 682733. 34421791 10.3389/fneur.2021.682733 PMC8371178

[14] Dammers R, Haitsma IK, Schouten JW, Kros JM, Avezaat CJ, Vincent AJ. Safety and efficacy of frameless and frame-based intracranial biopsy techniques. Acta Neurochir. 2008;150(1):23–9. 18172567 10.1007/s00701-007-1473-x

[15] D’Souza M, Gendreau J, Feng A, Kim LH, Ho AL, Veeravagu A. Robotic-Assisted Spine Surgery: History, Efficacy, Cost, And Future Trends. Robotic surgery (Auckland). 2019;6:9–23. 31807602 10.2147/RSRR.S190720 PMC6844237

[16] Wu Z, Chen D, Pan C, Zhang G, Chen S, Shi J, et al. Surgical Robotics for Intracerebral Hemorrhage Treatment: State of the Art and Future Directions. Ann Biomed Eng. 2023;51(9):1933–41. 37405558 10.1007/s10439-023-03295-x PMC10409846

[17] Wu S, Wang H, Wang J, Hu F, Jiang W, Lei T, et al. Effect of Robot-Assisted Neuroendoscopic Hematoma Evacuation Combined Intracranial Pressure Monitoring for the Treatment of Hypertensive Intracerebral Hemorrhage. Front Neurol. 2021;12: 722924. 34925205 10.3389/fneur.2021.722924 PMC8674426

[18] Heiden BT, Mitchell JD, Rome E, Puri V, Meyers BF, Chang SH, et al. Cost-Effectiveness Analysis of Robotic-assisted Lobectomy for Non-Small Cell Lung Cancer. Ann Thorac Surg. 2022;114(1):265–72. 34389311 10.1016/j.athoracsur.2021.06.090

[19] Singh R, Wang K, Qureshi MB, Rangel IC, Brown NJ, Shahrestani S, et al. Robotics in neurosurgery: Current prevalence and future directions. Surg Neurol Int. 2022;13:373. 36128120 10.25259/SNI_522_2022 PMC9479589

[20] Zhang JN, Liu JL. [Robotics Helps Usher in a New Era of Neurosurgery]. Sichuan Da Xue Xue Bao Yi Xue Ban. 2022;53(4):554–8. Chinese. 10.12182/20220760205 PMC10409464 35871722

